# Proteomic Contributions to Medicinal Plant Research: From Plant Metabolism to Pharmacological Action

**DOI:** 10.3390/proteomes5040035

**Published:** 2017-12-07

**Authors:** Akiko Hashiguchi, Jingkui Tian, Setsuko Komatsu

**Affiliations:** 1 Faculty of Medicine, University of Tsukuba, Tsukuba 305-8577, Japan; 2 College of Biomedical Engineering & Instrument Science, Zhejiang University, Hangzhou 310027, China; tjk@zju.edu.cn; 3 Faculty of Life and Environmental Sciences, University of Tsukuba, Tsukuba 305-8572, Japan; komatsu.setsuko.fu@u.tsukuba.ac.jp

**Keywords:** proteomics, medicinal plants, metabolic pathway, pharmacological action, accessibility to sequence data, access and benefit-sharing

## Abstract

Herbal medicine is a clinical practice of utilizing medicinal plant derivatives for therapeutic purposes. It has an enduring history worldwide and plays a significant role in the fight against various diseases. Herbal drug combinations often exhibit synergistic therapeutic action compared with single-constituent dosage, and can also enhance the cytotoxicity induced by chemotherapeutic drugs. To explore the mechanism underlying the pharmacological action of herbs, proteomic approaches have been applied to the physiology of medicinal plants and its effects on animals. This review article focuses on the existing proteomics-based medicinal plant research and discusses the following topics: (i) plant metabolic pathways that synthesize an array of bioactive compounds; (ii) pharmacological action of plants tested using in vivo and in vitro studies; and (iii) the application of proteomic approaches to indigenous plants with scarce sequence information. The accumulation of proteomic information in a biological or medicinal context may help in formulating the effective use of medicinal plants.

## 1. Introduction

Medicinal plants have been the long-standing backbone of herbalism worldwide and are an important part of biomedical innovation. Many therapeutic drugs used in conventional medicine were discovered on the basis of traditional knowledge of medicinal herbs, and were developed through multidisciplinary scientific validation [1]. One of the most important drug discoveries is the highly effective anticancer drug paclitaxel, which is naturally derived from *Taxus brevifolia* [2]. Although extensive screening of plant-derived natural products is underway, a vast number of plant components that can be promising drug sources remain unidentified [3]. Identifying such active compounds is difficult owing to multi-class secondary metabolites and the complex biosynthesis of these metabolites [4]. Biosynthesis of secondary metabolites is the summation of plant genetics and physiological responses to environmental factors. Considerable differences in the makeup of bioactive compounds such as flavonoids and saponins have been reported in a single species [5,6]. Moreover, most of the bioactive compounds in medicinal plants are extensively modified by chemical reactions. Multiple glycosylation steps form branch points within the biosynthetic pathways and lead to the production of diverse subfamilies of compounds with diverse drug-related properties [7,8]. Therefore, the understanding of the metabolic networks in medicinal plants and their systemic regulation is crucial for the future identification of active compounds.

Practical application of new drugs requires precise understanding of pharmacological properties of the compounds for their optimal use in clinical practice. Much attention has been paid to publishing scientific evidence for the effectiveness of plant-derived natural products [9]. In cancer therapy, various drug target pathways have been identified, including cyclin-mediated cell proliferation [10], p53-dependent apoptosis [11], and cytotoxicity accompanied with autophagy [12]. In addition to these pleiotropic antitumor effects, plant-derived products such as scutellarin, which is a type of flavone, can potentiate the effect of antitumor cytotoxic drugs, such as cisplatin [13]. Such herb–drug interactions can affect the outcome of pharmacotherapy in both favorable and unfavorable directions by influencing the enzymatic activity of drug metabolism in the body [14]. Furthermore, the occurrence of synergistic effects between compounds has been shown using different combinations of bioactive compounds isolated from *Citrus sinensis* [15]. The effect of mixed crude extracts from three herbs was also evaluated for potentiated effects [16]. One component in a multi-herbal formula may be identified as the active principle, but the mixture may include ingredients that cause potentiating or antagonistic effects, which are crucial for assessing the formula’s pharmacological action. Elucidating the mechanism underlying the pleiotropic action of herbal combinations can help understand the synergy between compounds and assist in developing suitable drugs with negligible adverse effects.

The complexity of drug discovery based on plant products requires a highly integrated approach with respect to medicinal plant science. In this regard, a proteomic approach for medicinal plant research is described in the following section to elucidate plant metabolic pathways that synthesize bioactive compounds. The subsequent section deals with responses of cultured cells and animal models treated with plant-derived bioactive compounds and extracts to understand the pharmacological action of the plants. In the final section, we surveyed an emerging field of proteomic application to indigenous plants in tropical regions. Accessibility to sequence data plays a key role in promoting the potential scientific value of indigenous and underutilized plants.

## 2. Plant Metabolic Pathways that Synthesize an Array of Bioactive Compounds

**Figure 1:**
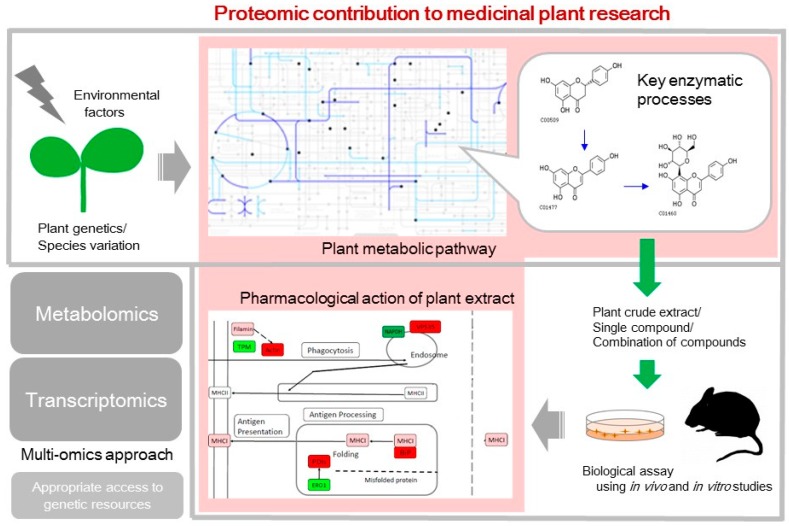
Overview of proteomic contribution to medicinal plant research. Proteomic technique, combined with other omics approaches, can shed light on both physiological aspects of plant secondary metabolism in response to environmental factors and pharmacological action of plant-derived active compounds on animals.

Plants utilize their metabolic systems for the biosynthesis of a wide variety of bioactive compounds to adapt to challenging ecosystems [17]. The metabolism of these compounds is influenced by plant genetics and environmental factors (Figure 1). Therefore, the composition and amount of bioactive compounds can exhibit intra- and inter-species variation [5,18]. Biological changes along with plant growth or in response to environmental stresses have been clarified in major crop species using proteomic techniques [19,20]. Proteins specifically relevant to the control of metabolic pathways accelerate the development of new cultivars. An example of this is folate-biofortified rice, which has provided health benefits in the developing world [21]. Metabolic engineering of multiple genes such as *GTP cyclohydrolase*, *ADC synthase*, together with either *folylpolyglutamate synthase* or *folate-binding protein* has been successfully implemented and has increased the folate content in folate-biofortified rice by over 100-fold than in non-engineered rice [22]. The introduction of genes of folate-synthesizing enzymes and folate-stabilizing factors enhances the folate content; this highlights the importance of requirement of thorough knowledge of the regulation of the endogenous metabolic pathways.

Proteomic techniques, which enable the measurement of systemic changes during cellular metabolism, have been used to analyze the synthesis of bioactive compounds that confer to medicinal plants their health promoting properties (Figure 1) [23]. *Cannabis sativa*, or the marijuana plant, is often used to reduce chronic pain [24]. Comparative proteomics was applied to identify specific tissue-expressed proteins of *C. sativa* because cannabinoids, the major active compounds of the plant, are found in many parts of the plant such as glands, flowers, and leaves [25]. *Panax ginseng* is a perennial herb that is used to treat physiological disorders such as diabetes, heart disease, cancer, and neurodegeneration [26]. The active components in ginseng include ginsenosides, a class of triterpene saponins that are found almost exclusively in *Panax* spp. [27]. Because the cultivation of ginseng is challenging owing to its long-life cycle [28], understanding the biosynthetic pathways of ginsenosides becomes crucial [29]. The metabolic changes along with plant growth were elucidated using proteomic analysis, revealing that ginseng roots initiate ginsenoside biosynthesis when the plant reaches a slow-growth period [30]. Such changes in root physiology demonstrate the early activity of enzymes that mediate upstream processes within the ginsenoside biosynthesis network and upregulation of enzymes that mediate downstream processes in later stage [31]. Recent proteomic analysis of a type of ginseng cultivar that grows slower than wildtype ginseng revealed that the cultivar increases ginsenoside biosynthesis as the plant ages [32]. The correlation of changes in energy metabolism with ginsenoside content was revealed by metabolic profiling [33]. In addition to traditionally used roots, the medicinal values of ginseng fruits were evaluated using proteomics [23]. A correlation between the abundance of the 6-phosphogluconate dehydrogenase family protein and radical scavenging activity of ginseng fruits was revealed by comparing different cultivars [23]. The results show the usefulness of proteomic techniques to discover key enzymes that are crucial for biological activities of medicinal plants. 

Improved and accurate identification of proteins via proteomics is available for various medicinal plants [34]. Jointly used with RNA sequencing, isobaric tags for relative and absolute protein quantitation (iTRAQ) helped identify four proteins belonging to the CYP718A subfamily of enzymes in *Anemone flaccida* that are essential for triterpenoid saponin biosynthesis [35]. The choice of sequence database is important for biosynthetic pathway analysis, as is indicated by a report that ascertained a series of enzymes playing a role in the final stages of artemisinin biosynthesis in *Artemisia annua* [36]. A metabolomic approach is another tool for the analysis of biosynthetic pathways for useful phytochemicals in plants [37]. Metabolomic analysis of *Salvia miltiorrhiza*, a perennial herb belonging to the *Lamiaceae* family, revealed differences of secondary metabolite profiles among several genotypes and phenotypes [38,39]. A wide array of secondary metabolites is synthesized through specific metabolic pathways that are often triggered by environmental stresses. A series of reports focusing on the effect of ultraviolet (UV) irradiation found that UV-A treatment of *Taxus chinensis*, a medicinal herb used against cancer, activated a photosynthetic reaction [40], and that the enhanced glycolysis provided precursors for secondary metabolism [40]. UV-B treatment of *Clematis terniflora*, which is a source of antitumor drugs, demonstrated the accumulation of indole alkaloids [41], upregulation of flavonoid biosynthesis [42], and increased γ-aminobutyric acid levels [43]. Enhanced flow of amino acids into the secondary metabolic pathways was shown by proteomic identification of enzymes like *S*-adenosylmethionine synthase and cysteine synthase [43]. Metabolic content in medicinal plants was improved by changes in abundance in 10-hydroxygeraniol oxidoreductase in *Catharanthus roseus* [44], or 1-deoxy-d-xylulose 5-phosphate reductoisomerase and 5-enol-pyruvylshikimate-phosphate synthase in *Lonicera japonica* [45]. UV-B irradiation can also cause the reduction of allergic compounds such as ginkgolic acids in *Ginkgo biloba* [46]. For the proteomic analysis of secondary metabolism in medicinal plants, purification of proteins that are low in abundance by application of combinatorial peptide ligand libraries (CPLL) or polyethylene glycol fractionation treatment was established as effective [45,47].

Agricultural proteomics is important in identifying useful plant cultivars, molecular mechanisms that underlie desirable physiological traits of the cultivars, and the response of plant cells to environmental stresses (Figure 1) [48]. For medicinal plants, these strategies are also effective in unraveling secondary metabolism networks and novel enzymes that remain unexplored (Figure 1).

## 3. Pharmacological Action of Plants Tested Using In Vivo and In Vitro Studies

Traditional medicine uses medicinal plants to treat chronic heterogeneous disorders worldwide, especially in developing countries [49]. As the population ages in developed countries, self-medication using herbal remedies has become a choice for promoting healthier living [50]. However, despite the long history of effective use of medicinal plants, many of them remain untested for efficacy and safety. In terms of identifying and evaluating active components in the extracts of medicinal plants, the beneficial and adverse effects of isolated compounds require to be tested against cultured cell lines (Figure 1).

Proteomics has been used to study cellular responses to bioactive compounds such as oridonin, a diterpenoid from *Rabdosia rubrescens*; curdione, a sesqiterpenoid from *Cucurma aromatica*; and geniposide, an iridoid glycoside from *Gardinia jasmonoides* [51,52,53]. Findings of a two-dimensional electrophoresis study identified stathmin as a target of R. rubrescens-derived oridonin in human hepatocarcinoma cells [51]. Because stathmin is a strong prognostic biomarker of poor survival in non-small cell lung cancer [54], oridinin has important implications in cancer therapy. With respect to antithrombotic activities of *C. aromatica*, proteins that are responsible for focal adhesion such as talin 1 and beta-1 tubulin were identified by gel-free proteomic techniques in curdione-treated human platelets, clarifying the molecular mechanism of action by *G. jasmonoides* [52]. For the detection of adverse side effects, biomarkers were searched for in geniposide overdose-induced hepatic injuries in rat model [53]. Using the intensity-based absolute quantification (iBAQ) method, glycine *N*-methyltransferase and glycogen phosphorylase were found to induce hepatotoxicity during the initial stages [53]. Subproteome-capturing technology unraveled how energy metabolism imbalance is caused in mitochondria to exert the anticancer activity of dihydroartemisinin from *Artemisia annua* [55]. Understanding of cellular responses is promoted by proteomic analyses in various cultured cell lines and animal models that are treated with natural products from medicinal plants.

It is increasingly evident that crude plant extracts containing complex mixtures of compounds often show greater activity than isolated constituents at an equivalent dose [56]. One report emphasized that total saponins are the active components of *Achyranthes aspera*, which can neutralize bone degeneration caused by rheumatoid arthritis in rats [57]. Total flavonoids isolated from *Scutellaria baicalensis* water extract show antimicrobial, antiviral, anti-inflammatory, and antitumor effects by inhibiting NF-κB signaling [58,59]. Moreover, studies that focused on the immunomodulatory effect of *Vigna radiata* water extract demonstrated that total flavonoids but not one of the flavonoids in the extract has the biological activity in murine cells [60]. Neither synthetic vitexin, which is the major flavonoid in *V. radiata*, nor its derivative can modulate interleukin-6 expression, but the removal of total flavonoids diminishes the effect of the extract on interleukin-6 expression [60]. Crude extract of *V. radiata* has a protective pleiotropic effect in mice against sepsis [61]. The pleiotropic effect was revealed to be caused by the plant’s ability of both immunosuppressing the acute inflammation and promoting antigen processing/presentation [60]. The search for its active main compound is under progress, but information regarding the composition of the *V. radiata* extract is present. Bioactive compounds such as prostaglandins and their biosynthetic pathways were identified in the species [5]. The comprehensiveness of proteomic profiling allowed the elucidation of the complex biological processes occurring in mice treated with medicinal plant extracts (Figure 1).

A traditional Chinese medicine prescription called Buyang Huanwu decoction, composed of seven kinds of medicinal plants, is used for treating cerebral ischemic stroke-induced disability [62]. It was shown to suppress the release of glutamate [63], increase the abundance of synapse-related proteins, and maintain the synaptic ultrastructure in cerebral ischemic rats [64]. The biological reaction against the treatment was elucidated by iTRAQ-based proteomic analysis [65]. In the mouse stroke model, the levels of proteins associated with stroke responses increased, including blood–brain barrier breakdown and enhanced excitotoxicity, but they were restored after treatment with the herb mixture. Functional profiling showed that the herb mixture treatment rescued disturbed energy metabolism and increased the neurogenesis marker doublecortin [65]. The report also showed the multiple effects of medicinal plants on ischemic stroke including the protective role and recovery against injury [65]. Buzhong Yiqi is a herbal formula used for treating various conditions [66] such as muscle atrophy, whereby the decoction can downregulate nuclear receptor corepressor 1 and its downstream factors [67]. Mitochondrial oxidative metabolism was identified as the mechanism underlying the Buzhong Yiqi-mediated recovery of muscle quantity [67]. Furthermore, the therapeutic mechanism underlying Shufeng Jiedu comprises eight medicinal herbs, and AKT1 kinase was considered as a key factor in acute lung injury [68]. The hypothesis from proteomic data was proved by gain- and loss-of-function analyses of macrophages, displaying that AKT1-mediated biological processes, including oxidative stress, apoptosis, and inflammatory responses were involved in acute lung injury [68]. Most medicinal plants are used for treating different symptoms. The identification of target pathways by proteomic profiling explains the various applications in heterogeneous disorders and will help improve the treatment regimen (Figure 1). On the other hand, efforts have been made to identify active compounds in the extracts of medicinal plants and validate their functionality. Narrowing the candidate list of active main compounds will also be possible using the identified pathways that are affected by the treatment as markers of physiological responses.

## 4. Application of Proteomics to Indigenous Plants

Plants that became the focus of attention of the above scientific studies were commercialized species. A protein catalogue was provided for the mango plant, which is a leading fruit crop and produces the unique xanthonoid called mangiferin [69,70]. A recent study improved protein purification methods for extracting proteins existing in small quantity in mango fruit pulp and banana [71,72]. In addition to the popular plants, some of the indigenous and underutilized plants were analyzed for their phytochemicals and physiology [73]. Proteomic profiles of plants such as *Nigella sativa*, *Moringa oleifera*, and *Pseudostellaria heterophylla* that are used in traditional medicine have been published [74,75,76]. Protein extraction was optimized for seagrass species *Zostera muelleri* and *Posidonia australis*, which contain hundreds of bioactive compounds and are used as cosmetic additives [77,78]. Proteomics is used for the efficacy evaluation of epigenetic intervention of carotenoid biosynthesis in *Zingiber zerumbet*, a tropical plant used as a tonic and a stimulant [79]. Contribution of proteomic techniques to medicinal plant science is not limited to elucidate the systemic changes in physiology; it is also used for isolating key enzymes in useful compound production. *Centella asiatica* is a herb used in Asian countries, which has become popular in the rest of the world [80,81]. Saponins are the active compounds in this plant that are modified by multiple glycosylation. In the search for saponin glucosyltransferases in *C. asiatica*, a successfully isolated enzyme was the triterpenoid carboxylic acid: UDP-glucose 28-*O*-glucosyltransferase [82]. Many unique enzymes and metabolic pathways can be discovered by appropriate application of proteomic techniques.

Constraints associated with medicinal plant studies include the complexity of plant secondary metabolism, which is often unique to the species, and the lack of genomic information. Genome sequences have been provided for some species [83,84], but as the basis for secondary metabolism studies, transcriptomic analysis is of growing importance (Figure 1) [85]. RNA sequencing-based transcriptomic data are now available for medicinal plants such as *Dendrobium officinale* [86], *Dioscorea nipponica* [87], *Ephedra sinica* [88], *L. japonica* [89], and *Polygonum minus* [90]. Not only medicinal plants, but also other tropical plants, were studied by RNA sequencing-based transcriptomic analysis with an eye for understanding the unique characteristics of diverse plants such as pitcher plants [91,92]. The high throughput sequences obtained using RNA sequencing are archived in public data repositories such as the sequence read archive so that the data can be reanalyzed [93].

Data sharing and comparison are crucial for promoting scientific work in the biological sciences. Today, over 4400 research activities are supported by the research infrastructure Kyoto Encyclopedia of Genes and Genomes (KEGG), which provides cross-organismal annotation of genes [94]. However, there is concern for open-access utilization of high throughput sequences from the viewpoint of access and benefit-sharing (ABS), which refers to the rules governing the use of genetic resources and associated traditional knowledge. Attention must be paid to the international rules and appropriate access to genetic resources to promote responsible conduct of medicinal plant research.

## 5. Conclusions

Medicinal plants are a potential source of new drugs and therapeutics. However, many natural products and biosynthetic pathways, which can be promising sources for medical applications, remain unidentified. Pharmacological activities of many medicinal plants are not fully elucidated. Two directions for future research on medicinal plants, which are plant physiological studies especially on secondary metabolism and pharmacological actions of plant-derived compounds on animals (Figure 1), are necessary for proper and sustainable utilization of plant genetic resources and their metabolic products. Proteomics offers the advantage of collating a comprehensive collection of physiological information both from medicinal plants and animals that consume the plants. The identification of key enzymes has helped elucidate the multistep, complicated biosynthetic pathways of plant bioactive compounds. External factors that affect the pathways and the physiological changes caused by the factors are described using the proteomic analyses of plants. Responses of the animals treated with plant-derived natural products were shown to include various biological processes. These studies are two sides of the same coin in research on medicinal plants. Combining these research disciplines by increasing instances of multidisciplinary collaborative research that involve researchers from both fields would help promote research on medicinal plants.

## Abbreviations

iTRAQ	isobaric tags for relative and absolute protein quantitation
UV	ultraviolet
CPLL	combinatorial peptide ligand libraries
iBAQ	intensity-based absolute quantification
NF-κB	nuclear factor-kappa B
AKT1	RAC serine/threonine-protein kinase
ABS	access and benefit-sharing
